# Correlation of serum ferritin, D dimer and CRP levels with disease severity among hospitalized patients in India

**DOI:** 10.6026/973206300223572

**Published:** 2026-06-30

**Authors:** Madhur Gupta, Girish Dubey, Ritesh Yadav, Ramesh Prasad Agrawal

**Affiliations:** 1 Department of Biochemistry, N.K.P. Salve Institute of Medical Sciences & Research Centre and Lata Mangeshkar Hospital, Nagpur, Maharashtra, India; 2 Department of General Medicine, SRVS Medical College Shivpuri, Madhya Pradesh, India; 3 Department of Microbiology, Government Medical College, Satna, Madhya Pradesh, India

**Keywords:** Serum ferritin, C-reactive protein (CRP), D-dimer, disease severity, inflammatory biomarkers, hospitalized patients

## Abstract

Delayed recognition of severe disease in hospitalized patients remains a major challenge and contributes to increased morbidity and mortality. Therefore, it is of interest to evaluate the correlation of serum ferritin, C-reactive protein (CRP) and D-dimer levels with disease severity in hospitalized patients. Hence, a hospital-based cross-sectional study was conducted on 100 adult patients and biomarker levels were measured using standard laboratory methods. The results showed a significant increase in serum ferritin, CRP and D-dimer levels with increasing disease severity (p < 0.001). A strong positive correlation was observed between these biomarkers and disease severity, with D-dimer showing the highest correlation and elevated levels were significantly associated with ICU admission and mortality. Thus, combined assessment of ferritin, CRP and D-dimer is a simple and reliable approach for early risk stratification and prognostication in hospitalized patients.

## Background:

Assessment of disease severity in hospitalized patients is essential for timely management and improved outcomes. Inflammatory and coagulation biomarkers have gained significant importance as reliable indicators of disease progression and prognosis [1]. C-reactive protein (CRP) is an acute-phase reactant that reflects systemic inflammation and correlates with disease severity and adverse clinical outcomes. Similarly, serum ferritin, beyond its role in iron storage, acts as a marker of hyperinflammation and immune dysregulation, with elevated levels associated with severe disease states [2, 3]. D-dimer, a fibrin degradation product, indicates activation of coagulation pathways and is linked to thrombotic complications and increased mortality. The interaction between inflammation and coagulation (thromboinflammation) plays a critical role in disease progression [4]. Recent studies suggest that combined evaluation of CRP, ferritin and D-dimer provides better risk stratification compared to individual markers [3, 4, 5]. However, variability exists across populations, necessitating further research. Therefore, it is of interest to evaluate the correlation of these biomarkers with disease severity in hospitalized patients.

## Materials and Methods:

This was a hospital-based observational cross-sectional study conducted in the Department of General Medicine at a tertiary care teaching hospital over a period of one year (from January 2025 to December 2025). A total of 100 adult patients admitted to the hospital with clinically diagnosed acute or chronic illness requiring hospitalization were considered for inclusion

## Inclusion criteria:

[1] Patients aged ≥18 years

[2] Patients requiring hospitalization for medical illness

[3] Patients who provided informed consent

## Exclusion criteria:

[1] Patients with known hematological malignancies

[2] Patients on anticoagulant therapy prior to admission

[3] Patients with chronic liver disease or known iron metabolism disorders

[4] Pregnant women

[5] Patients with incomplete laboratory data

## Data collection:

Detailed clinical history, demographic details and relevant examination findings were recorded using a pre-designed structured proforma.

## Assessment of disease severity:

Patients were categorized into mild, moderate and severe disease groups based on:

[1] Clinical presentation

[2] Requirement of oxygen/ventilatory support

[3] ICU admission

[4] Organ dysfunction parameters

## Laboratory investigations:

Venous blood samples were collected under aseptic conditions at the time of admission.

The following parameters were measured:

[1] Serum Ferritin (ng/mL): Measured using chemiluminescent immunoassay

[2] C-reactive Protein (CRP) (mg/L): Measured using immunoturbidimetric method

[3] D-dimer (ng/mL or μg/mL): Measured using ELISA/latex-enhanced immunoassay

All investigations were carried out in the central clinical laboratory following standard protocols.

Correlation of serum ferritin, CRP and D-dimer levels with disease severity was measured

## Statistical analysis: 

Data were entered into Microsoft Excel and analyzed using SPSS version 25.0. Continuous variables were expressed as mean ± standard deviation (SD) whereas categorical variables were expressed as frequency and percentage. Comparison between groups was done using ANOVA / Student's t-test for continuous variables and Chi-square test for categorical variables. A p-value <0.05 was considered statistically significant

## Results:

A total of 100 hospitalized patients were included in the study. Out of the total 100 patients, the majority belonged to the 41-60 years age group (42%), followed by patients aged >60 years (30%) and 18-40 years (28%). There was a male predominance, with 62% males and 38% females included in the study (Table 1). Among the study population, 38% patients had moderate disease, 34% had mild disease and 28% were categorized as severe, indicating a relatively higher proportion of patients in the moderate severity group (Figure 1 - see PDF). The mean levels of all three biomarkers-serum ferritin, CRP and D-dimer-showed a progressive increase from mild to severe disease (Table 2). A strong positive correlation was observed between biomarker levels and disease severity (Table 3). Patients requiring ICU admission and those who expired had significantly higher biomarker levels compared to others (Table 4).

## Discussion:

In this study, the majority of patients belonged to the middle and older age groups, with a male predominance. Similar demographic patterns have been reported in recent studies, suggesting that advanced age and male gender are associated with increased disease severity and worse outcomes, possibly due to altered immune responses and higher comorbidity burden [6, 7]. A key finding of the present study was the significant increase in CRP levels with disease severity. CRP is a well-established acute-phase reactant and reflects the intensity of systemic inflammation. Our findings are consistent with recent studies demonstrating that elevated CRP levels are associated with severe disease and poor clinical outcomes [7, 8]. A recent study (2024) reported significantly higher CRP levels in severe cases, indicating its role as a reliable marker of inflammation and disease progression. Furthermore, a meta-analysis showed that elevated CRP is significantly associated with severe disease and adverse outcomes, reinforcing its prognostic value [9]. Serum ferritin levels in our study also showed a marked increase with disease severity, supporting its role as a marker of hyperinflammation. Hyperferritinemia is increasingly recognized as an indicator of cytokine storm and macrophage activation. A recent study demonstrated a significant positive correlation between ferritin levels and disease severity, which is in agreement with our findings. Additionally, elevated ferritin levels have been associated with increased mortality and severe clinical outcomes, further emphasizing its prognostic significance [10]. D-dimer levels in the present study showed the strongest correlation with disease severity and were significantly elevated in severe cases, ICU admissions and non-survivors. This finding highlights the role of coagulation abnormalities and thromboinflammation in disease progression. Similar observations have been reported in recent literature, where elevated D-dimer levels were strongly associated with disease severity, ICU admission and mortality. The meta-analysis by Huang *et al.* [9], also demonstrated that increased D-dimer levels are linked with a higher risk of severe disease and mortality. The present study also found that patients requiring ICU admission and those who expired had significantly higher levels of all three biomarkers, indicating their usefulness in predicting poor outcomes. These findings are supported by recent studies showing that elevated CRP, ferritin and D-dimer levels are associated with increased need for intensive care and higher mortality rates [11, 12]. Another important observation in our study was the strong positive correlation between biomarkers and disease severity, suggesting that these markers reflect underlying pathophysiological mechanisms such as systemic inflammation, immune dysregulation and coagulation activation. Recent studies have emphasized that combined biomarker evaluation provides better prognostic accuracy compared to individual markers, as it captures multiple aspects of disease pathology [11, 13].

## Conclusion:

Serum ferritin, CRP and D-dimer levels increase significantly with disease severity and show a strong positive correlation, with D-dimer being the strongest predictor. Elevated levels are also associated with ICU admission and mortality. Thus, combined assessment of ferritin, CRP and D-dimer serves as a simple, cost-effective and reliable tool for early risk stratification and prognostication in hospitalized patients. Incorporating these biomarkers into routine clinical evaluation may aid in timely identification of high-risk patients and improved clinical decision-making.

## Figures and Tables

**Table 1:** Demographic characteristics of study population (n = 100)

**Variable**	**Number (%)**
Age (years)	
18-40	28 (28%)
41-60	42 (42%)
>60	30 (30%)
**Gender**	
Male	62 (62%)
Female	38 (38%)

**Table 2:** Mean levels of biomarkers according to disease severity

**Biomarker**	**Mild (n=34)**	**Moderate (n=38)**	**Severe (n=28)**	**p-value**
Serum Ferritin (ng/mL)	210 ± 85	420 ± 130	780 ± 210	<0.001
CRP (mg/L)	12.5 ± 6.2	36.8 ± 12.4	82.3 ± 25.6	<0.001
D-dimer (ng/mL)	320 ± 140	890 ± 310	1850 ± 620	<0.001

**Table 3:** Correlation of biomarkers with disease severity

**Biomarker**	**Correlation Coefficient (r)**	**p-value**
Serum Ferritin	0.68	<0.001
CRP	0.72	<0.001
D-dimer	0.75	<0.001

**Table 4:** Association of biomarkers with clinical outcomes

**Outcome**	**Ferritin (Mean ± SD)**	**CRP (Mean ± SD)**	**D-dimer (Mean ± SD)**	**p-value**
ICU Admission (n=30)	720 ± 200	78 ± 24	1700 ± 580	<0.001
No ICU (n=70)	310 ± 120	28 ± 15	620 ± 250	
Mortality (n=18)	810 ± 230	88 ± 26	1950 ± 640	<0.001
Survivors (n=82)	360 ± 140	32 ± 18	720 ± 300	
